# High-resolution mapping of brain vasculature and its impairment in the hippocampus of Alzheimer's disease mice

**DOI:** 10.1093/nsr/nwz124

**Published:** 2019-08-28

**Authors:** Xiaochuan Zhang, Xianzhen Yin, Jingjing Zhang, Anan Li, Hui Gong, Qingming Luo, Haiyan Zhang, Zhaobing Gao, Hualiang Jiang

**Affiliations:** 1 School of Pharmacy, East China University of Science and Technology, Shanghai 200237, China; 2 CAS Key Laboratory of Receptor Research, Shanghai Institute of Materia Medica, Chinese Academy of Sciences, Shanghai 201203, China; 3 Britton Chance Center for Biomedical Photonics, Wuhan National Laboratory for Optoelectronics-Huazhong University of Science and Technology, Wuhan 430074, China; 4 MoE Key Laboratory for Biomedical Photonics, School of Engineering Sciences, Huazhong University of Science and Technology, Wuhan 430074, China; 5 Shanghai Institute for Advanced Immunochemical Studies, and School of Life Science and Technology, ShanghaiTech University, Shanghai 200031, China

**Keywords:** Micro-Optical Sectioning Tomography, image optimization, Alzheimer's disease, impaired hippocampal vasculature, virtual vascular endoscopy

## Abstract

Accumulating evidence indicates the critical importance of cerebrovascular dysfunction in the pathogenesis of Alzheimer's disease (AD). However, systematic comparative studies on the precise brain vasculature of wild-type and AD model mice are still rare. Using an image-optimization method for analysing Micro-Optical Sectioning Tomography (MOST) data, we generated cross-scale whole-brain 3D atlases that cover the entire vascular system from large vessels down to smallest capillaries at submicron resolution, for both wild-type mice and a transgenic (APP/PS1) mouse model of AD. In addition to distinct vascular patterns in different brain regions, we found that the main vessels of the molecular layer of the hippocampal dentate gyrus (DG-ml) undergo abrupt changes in both diameter and branch angle, spreading a unique comb-like pattern of capillaries. By using a quantitative analysis workflow, we identified in the hippocampus of AD mice an overall reduction of the mean vascular diameter, volume fraction and branch angle, with most significant impairment in the DG-ml. In addition, virtual endoscopy revealed irregular morphological features in the vessel lumen of the AD mice, potentially contributing to the impairment of blood flow. Our results demonstrate the capability of high-resolution cross-scale evaluation of brain vasculature and underscore the importance of studying hippocampal microcirculation for understanding AD pathogenesis.

## Introduction

Alzheimer's disease (AD) is a severe neurodegenerative disease with a high prevalence [1], but no treatment is currently available for modifying AD progression [2,3]. Although underlying mechanisms that drive the pathogenesis of AD remain to be fully elucidated [4,5], mounting evidence suggests that the structural and functional integrity of the cerebral vasculature is associated with both AD progression and severity [6]. Cerebral hypoperfusion began many years before the appearance of overt cognitive symptoms in AD [7–9], suggesting that cerebral blood flow (CBF) could be used as a predictor for AD development. Cerebrovascular lesion could cause insufficient supplies of glucose and other nutrients and impede the removal of harmful substances from lesion-inflicted brain regions. Middle-cerebral-artery occlusion resulted in a rapid increase in amyloid plaques in the region surrounding the infarction [10] and compromised cerebral vessels also led to impaired Aβ clearance [11]. In addition, cerebral vascular lesions could induce impairment of neurovascular regulation [12], particularly cholinergic innervation [13] and the breakdown of the blood–brain barrier (BBB) [14]. These vascular deficits eventually alter blood supply in the brain, leading to neuronal injury and cognitive impairment [15]. The cerebrovascular dysfunction in AD may also explain the low clinical efficacy in some pharmacological treatments of AD.

Substantial efforts have been made to uncover the relationship between AD and vascular abnormalities [16,17]. In AD model mice, the vascular amyloid could displace astrocyte end-feet from the endothelial vessel wall [12] and tau pathology could affect vascular endothelial cells [18], both altering the integrity of the cerebral microvasculature. Furthermore, pericyte degeneration was found to disrupt white-matter microcirculation, leading to white-matter functional deficits in AD mice [19]. These findings suggest that pathogenic factors of AD may impair basic cellular elements of the vasculature and disrupt the integrity of the BBB. Using wild-type and AD model mice, we further examined the structure of the brain vasculature at the mesoscopic level with subcellular resolution, in order to identify in detail changes of brain vasculature associated with AD.

The primary goal of this study is to obtain 3D atlases of the vasculature for macroscopic whole-brain samples at submicron resolution. High image resolution and large sample size are mutually exclusive in conventional imaging methods, where access to detailed images at the submicron resolution will restrict the detection range to the millimeter level. Thus, previous imaging studies were limited to either macro-vessels of the whole brain [20,21] or microvessels in a small brain region [22,23]. Micro-Optical Sectioning Tomography (MOST) [24] enables whole-brain imaging at the submicron level and had been used to provide the first mesoscopic description of the entire vasculature of the mouse brain [25]. In this study, we further developed the imaging-optimization method for the analysis of MOST data that allows direct visualization of somata and nerve processes, blood vessels down to capillaries and morphological features within the lumen of individual vessels. Using this method, we obtained mesoscopic atlases of the whole-brain vasculature for APPswe/PSEN1dE9 (APP/PS1) transgenic (Tg) (hereinafter referred to as Tg-AD) mice and wild-type (WT) mice. Furthermore, we quantitatively compared the hippocampal vasculature of these two types of mice, and identified several AD-related abnormalities. Given the important role of the hippocampus in learning and memory, these findings of the hippocampal abnormalities are likely to be causally related to the memory impairment in AD patients. Quantitative characterization of the vasculature abnormality may facilitate the diagnosis of AD progression and the development of vasculature-targeting therapeutic approaches.

## Results

### Acquisition of high-quality whole-brain atlases

**Figure 1.**
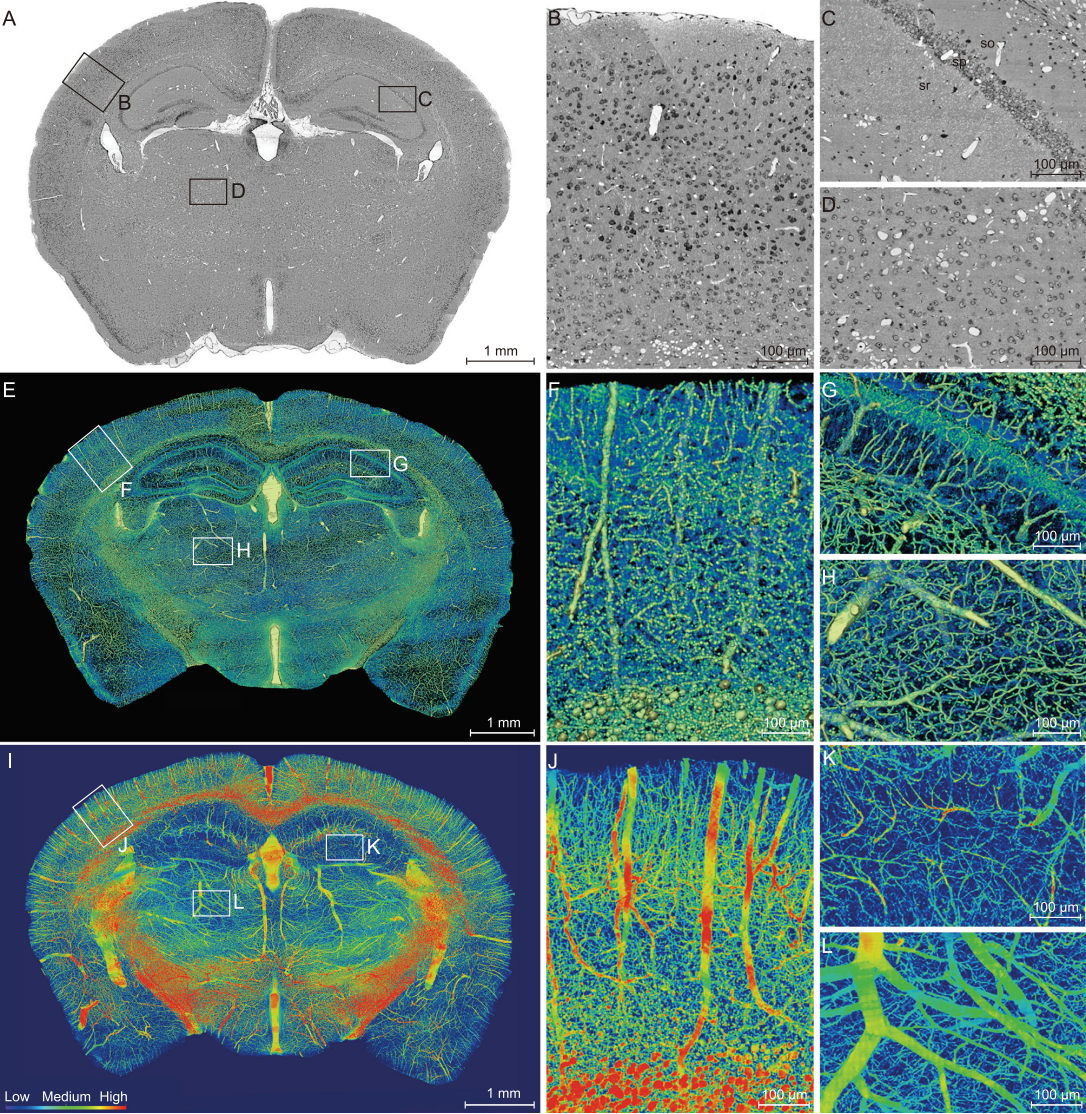
High-resolution brain atlas providing a global view of the complex vascular network in a C57BL/6 mouse. (A) Representative coronal section showing the cortex (B), hippocampus (C) and thalamus (D), using the whole-brain Nissl-staining method. Boxed areas in A are shown in B, C and D, revealing somata (black) and vessels (white). The *stratum oriens* (so), *strata pyramidale* (sp), *strata radiatum* (sr), cell layers in the hippocampus. Smaller white circles in sr, nerve processes. (E–H) An image of a 200-μm-thick coronal section of the mouse brain reconstructed using volume rendering, with gray levels color-coded. Boxed areas in the cortex F, hippocampus G and thalamus H are shown at a higher magnification. Yellow/green lines, blood vessels; green dots in *sp*, somata; radial blue lines in *sr*, dendrites. (I–L) Maximum intensity projection (MIP) of the 400-μm-thick coronal section adjacent to that shown in E, revealing only blood vessels, with the vascular volumetric density coded in color. Boxed areas of the cortex (J), hippocampus (K) and thalamus (L) are shown at a higher magnification.

Whole-mouse-brain datasets were acquired at a resolution level of 0.35 × 0.35 × 1.00 μm from 2-month-old male C57BL/6 mice. After completion of whole-brain Nissl staining and an image preprocessing as previously described [26], we found that the coronal images showed periodic strip noise and non-uniform brightness (Supplementary Fig. 1A). An image-optimization method (see the Methods section) was used to obtain high-quality images with clear cytoarchitecture and vasculature (Fig. 1A–D). In comparison with those obtained by the previous image-preprocessing method (Supplementary Fig. 1A and B), the gray value of the slice background became homogeneous and the whole range of the gray level was enlarged (Supplementary Fig. 1C and D), leading to a marked improvement in the background uniformity, noise reduction and image contrast (Supplementary Fig. 1E and F). A representative image of the coronal section is shown in Fig. 1A. The somata (in black) and vessels (in white) in the cortex (Fig. 1B), hippocampus (Fig. 1C) and thalamus (Fig. 1D) could be clearly identified. This optimization method enables complete visualization of the brain with all information and details preserved.

MOST can provide information regarding somata and vessels simultaneously. In addition, cross-sections of individual dendrites in the *stratum radiatum* of CA1 to CA3 could also be discerned as tiny circles (Fig. 1C). These dendrites appeared with a radial pattern and were mostly perpendicular to the pyramidal cell layer in 3D view (Fig. 1E and G), with the location and distribution consistent with the neural-connection map demonstrated by the Golgi-staining method and fluorescent protein-labeling technique [27]. Moreover, the gray value of these circles was lower than that of the vessels and higher than that of the somata (Supplementary Fig. 1C and D). Therefore, these data provided by MOST comprised three types of information, including somata, nerve processes and blood vessels.

### Visualization and characterization of the complex vascular network

To further visualize the global brain vasculature, we obtained 3D reconstruction of the vascular network from the sections of 2-month-old C57BL/6 mice. The volume rendering of the 200-μm-thick coronal sections revealed 3D images of cells, vessels and nerve processes, with gray levels coded by pseudo-colors. The enlarged view in Fig. 1E shows the compact vascular network in the cortex (Fig. 1F), hippocampus (Fig. 1G) and thalamus (Fig. 1H). In the high-resolution image of the hippocampus (Fig. 1G), the radial distribution of dendrites (blue) in the *stratum radiatum*, the vessels (yellow/green) and the somata in the pyramidal cell layer (deep green) can all be discerned.

We subsequently adopted a heat-map visualization of the vascular network in the 2-month-old C57BL/6 mice, with the density of the vessels coded

by a color scale and deep blue representing no vessels. The heat map of a representative 400-μm-thick coronal slice (Fig. 1I) showed distinct vascular distribution and density in the cortex (Fig. 1J), hippocampus (Fig. 1K) and thalamus (Fig. 1L). The cortical parenchyma was full of abundant vessels, which were largely arranged in parallel and penetrated the deep cortical layer, and the trunk vessels sent off small branches along the way (Fig. 1I and J). With respect to the hippocampal vasculature, equally spaced transverse hippocampal vessels entered the hippocampus perpendicularly to the coronal plane (Fig. 1I). The thalamus did not exhibit the same organized vascular network as that found in the hippocampus and cortex (Fig. 1I and L). The substantial deep-blue color in the hippocampal region indicates a low density of the vascular network in the hippocampus (Fig. 1I and K), as compared to other brain regions. The quantitative data on the vasculature of the three brain regions (Table 1) show that the mean vascular diameter, length density (total vascular length per tissue volume) and volume fraction (total vascular volume relative to the total tissue volume) were the lowest in the hippocampus. By contrast, the thalamus exhibited the highest mean vascular diameter and volume fraction, whereas the length density was lower than that in the cortex. The apparent paradoxical parameters in the thalamus might be caused by the large mean diameter of the thalamic vessels. The low vascular diameter, length density and volume fraction of the hippocampus suggest that the hippocampus is more vulnerable to cerebrovascular dysfunction.

**Table 1.** Parameters of vascular morphology in three areas of wild-type mouse brain.

	Mean diameter (μm)	Length density (m/mm^3^)	Volume fraction (%)	Maximum diameter (μm)
Cortex	2.13 ± 0.05	2.09 ± 0.05	1.19 ± 0.07	28.57 ± 1.28
Hippocampus	1.71 ± 0.09*^###^	1.69 ± 0.07**	0.92 ± 0.17*^#^	30.02 ± 0.90^#^
Thalamus	3.09 ± 0.11	1.77 ± 0.11	2.21 ± 0.36	45.86 ± 3.50

Note: Data are based on measurements from three ROIs of each brain region in a 2-month-old C57BL/6 mouse. The ROIs were selected similarly to those shown in Fig. 1F–H. Data were presented as mean ± s.e.m. (unpaired *t*-test; *p < 0.05; **p < 0.01 vs cortex; ^#^*p* < 0.05; ^###^*p* < 0.001 vs thalamus).

### Pattern of vasculature in the segmented normal hippocampus

**Figure 2.**
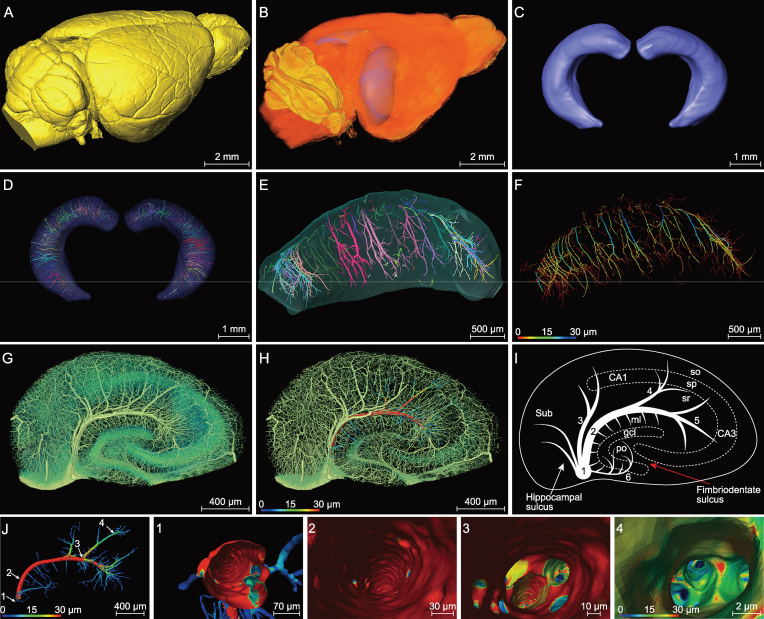
Segmented hippocampus for high-resolution vasculature analysis in a C57BL/6 mouse. (A) Lateral view of the 3D-reconstructed whole mouse brain, with a network of pial blood vessels visible on the surface. (B) and (C) Two segmented hippocampi from the mouse brain shown in (A), showing the location in the brain and the anterior view, respectively. (D) and (E) The inner vasculature of the bilateral hippocampi showing equally spaced transverse hippocampal vessels distributed in a rake-like pattern. Individual vessels labeled by different colors. (F) The same hippocampus as that in (E), with vessel diameters coded by colors. (G–I) Images of a hippocampal section perpendicular to the longitudinal axis, showing branches of transverse hippocampal vessels, with the gray level coded by the color in (G) (revealing both vessels, somata and nerve processes in *sr*). In (H), a selected vessel with the diameter of its main trunk and branches was coded for its diameters in colors (mainly vessels only). (I) Schematic diagram depicting the pattern of the transverse hippocampal vessels. 1: LHV, giving off ITHVs and ETHVs; 2: ITHA, entering the *cornu ammonis* by the hippocampal sulcus; 3: the branches directly generating from LHV to supply CA1; 4/5: the branches of ITHV respectively supplying CA2 and CA3; 6: ETHV, entering the dentate gyrus by penetrating the fimbriodentate sulcus. In *cornu ammonis*: so, *stratum oriens*; sp, *stratum pyramidale*; sr, *stratum radiatum*. In dentate gyrus: ml, molecular layer; po, polymorph layer; gcl, granule cell layer. (J) Representative individual hippocampal vessel the same as that shown in (H) was manually segmented, with vessel diameters coded by colors. The transverse hippocampal vessel appears like an arch with microvessels extending from its both dorsal and ventral sides. (1–4) The virtual vascular endoscopic tracking of the individual transverse hippocampal vessel. (1) Initial entry into the vessel. Subsequent images along the tracking pathway are shown in (2)–(4), revealing endovascular structures. The scale bar in each endoscopic image indicates the magnification of field of view.

Given the pivotal roles of the hippocampus in AD pathogenesis [28] and the distinct hippocampal vasculature described above, we manually segmented the hippocampus from the whole mouse brain (Fig. 2A and B). Manual segmentation is currently considered to be the most reliable segmentation method [29], as compared to atlas-based automatic segmentation methods [30]. In particular, manual segmentation is required for edge detection due to the low contrast between the region of interest and its neighboring structures in the Nissl-staining tissue. Figure 2B and C show the segmented hippocampus of the mouse brain. The procedure of whole-brain imaging and segmentation of hippocampus is shown in Supplementary Movie 1.

In view of the importance of the vasculature, a detailed description of the orientation and distribution of blood vessels in the hippocampus is useful for understanding the pathological mechanism of the vascular lesion underlying brain disorders. As shown in Fig. 2D–F, the vascular network of the segmented hippocampus exhibited a rake-like pattern, comprising a series of consistently spaced transverse hippocampal vessels. The main blood vessels of the hippocampus (LHV) ran largely in parallel with the longitudinal hippocampal axis and sent off internal and external transverse hippocampal vessels (including arteries and veins). The internal transverse hippocampal vessels (ITHVs) were located in the hippocampal sulcus and sent their arch-like branches to CA1, CA2 and CA3 areas, while external transverse hippocampal vessels (ETHVs) entered the DG by penetrating into the fimbriodentate sulcus (Fig. 2G–I). Additional arteries and veins arose from the LHVs or ITHVs to supply the subiculum (Sub) and CA1 fields (Fig. 2G–I). The ETHVs, which appeared shorter in length, smaller in diameter and did not supply as much tissue as ITHVs, extended their branches mainly to the hilar structures and infra-pyramidal blade of the molecular layer of dentate gyrus (DG-ml) (Fig. 2G–I). The capillaries formed a highly interconnected microvascular network in the distal end (Fig. 2G and H). Note that the small vessels infusing into the supra-pyramidal blade of the DG-ml directly originated from ITHVs rather than ETHVs, which mainly provided blood supply to the infra-pyramidal blade of the DG-ml. As illustrated by the diameter map of a single transverse hippocampal vessel (Fig. 2J), vessels supplying the DG-ml were all <10 μm, while those in other hippocampal subareas contained larger vessels. They also appeared shorter in length and were densely distributed perpendicularly to the transverse hippocampal vessels. The distinct vascular distribution and abrupt changes in both diameter and branch angle resulted in a unique comb-like capillary pattern. This vascular pattern is distinct from that found in other hippocampal sub-regions, where gradual hierarchical branching of the vessels was observed.

**Figure 3.**
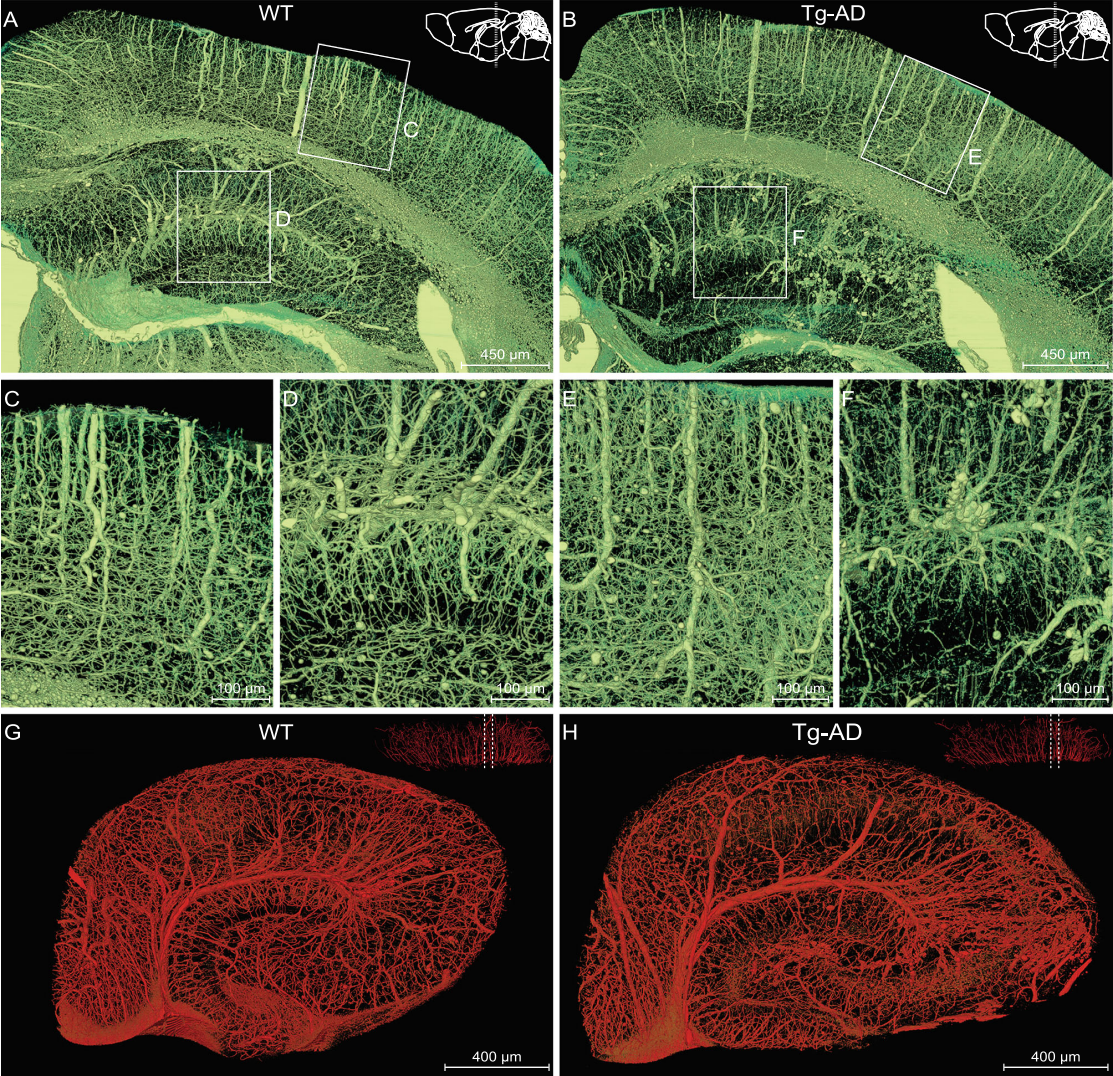
Comparison of the hippocampal vasculature between WT and Tg-AD mice. (A), (C), (D) and (G) WT group; (B), (E), (F) and (H) Tg-AD group. (A) and (B) Representative coronal view of the vascular network of the right hippocampus and its neighboring brain regions in WT and Tg-AD mice. The brain contours in the top-right corner indicate that the coronal slices between the two dotted lines were reconstructed. Boxed areas in the cortex ((C) and (E)) and hippocampus ((D) and (F)) are shown at a higher magnification. (G) and (H) Representative sectional views of the hippocampal vasculature perpendicular to the longitudinal hippocampal axis. The dotted lines in the top-right corner denote the data displayed in sectional view.

Figure 2J shows a single vascular branch extracted from Fig. 2H. We further performed the virtual vascular endoscopy of this single branch to explore the endoluminal landscape of the blood vessels. Fig. 2J (1–4) shows endoscopic images from the initiation to termination of the vessel (at the end of Supplementary Movie 1). The color-map in the endoscopic images reflects the size of the vascular lumen. This vascular endoscopy offered interesting information, including the inner-surface

morphology and smoothness of the vascular lumen and bifurcation pattern from a unique endovascular perspective. These endoscopic images showed that the inner surface of the C57BL/6 (WT) mouse was smooth and the trunk gave off branches in an orderly way. Moreover, we found that vessels ranging from 2 to 30 μm in diameter could all be visualized, allowing detailed examination of the endovascular morphology for both vascular trunks and capillaries.

**Figure 4.**
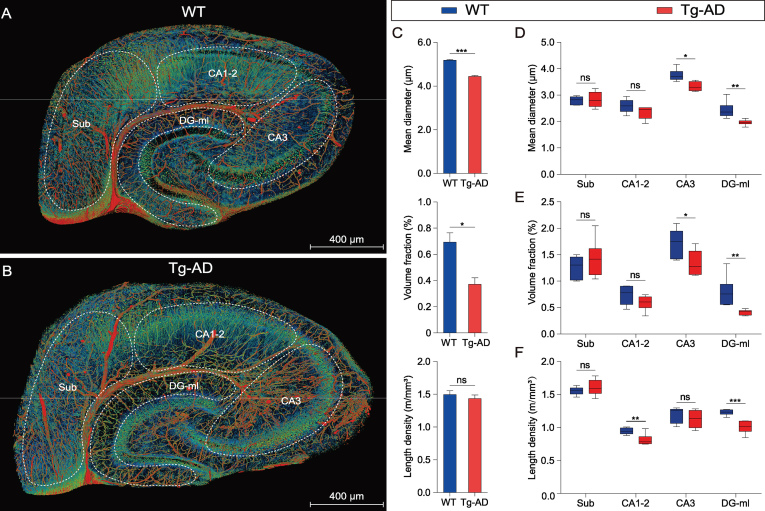
Quantitative comparison of the hippocampal vasculature between WT and Tg-AD mice. (A) and (B) The same sections as shown in Fig. 3G and H are displayed with somata, vessels and nerve processes together to facilitate the demarcation of hippocampal sub-regions. The hippocampal vasculatures were divided into four subareas based on the hippocampal functional partition including the subiculum (Sub), CA1–2, CA3 and molecular layer of the dentate gyrus (DG-ml). The quantitative analyses of the main morphometric parameters were conducted on the basis of these segmented sub-regions. (C–F) Quantitative analysis of the hippocampal vascular network. Columns and boxes in blue are the WT group; columns or boxes in red are the Tg-AD group. (C) Comparison of the mean diameter, volume fraction and length density in the whole hippocampus between WT and Tg-AD mice (unpaired *t*-test; *P < 0.05; ***P < 0.001; ns, non-significant, *P* > 0.05; *n* = 3 mice per group). (D–F) Comparison of the mean diameter, volume fraction and length density in the four segmented sub-regions between the WT and Tg-AD mice (unpaired *t*-test; *P < 0.05; **P < 0.01; ***P < 0.001; ns, non-significant, *P* > 0.05; *n* = 2 sections per mouse for three WT mice and three Tg-AD mice). Error bars in (C) represent s.e.m.; error bars in (D–F) represent the greatest value (above) and lowest value (below).

### Abnormalities of the hippocampal vasculature in Tg-AD mice

To determine whether the vasculature was impaired in AD mice, we performed a comparative analysis of the vasculature between Tg-AD mice and WT littermates. By viewing only vasculature

images (extraction based on gray values; see Methods), found clear vasculature abnormality in both cortex and hippocampus (Fig. 3A and B). The vessels appeared to be thinner and less uniform and organized in Tg-AD mice, as compared to those in the WT mice (Fig. 3C–F). To investigate the vasculature impairment in the hippocampus, we segmented the right hippocampus to perform volume rendering of the vessels and obtained representative views of cross-sections perpendicular to the longitudinal hippocampal axis (Fig. 3G and H; see Supplementary Movie 2), showing clearly disorganized vessels in the Tg-AD hippocampus.

Figure 4A and B depict images of the same sections as shown in Fig. 3G and H over a wider range of gray levels (color-coded) that revealed neuronal cell bodies and nerve processes, in addition to the vasculature, allowing identification of different subareas of the hippocampus. Quantitative analysis of the vasculature parameters was performed for the entire right hippocampus from three WT mice and three Tg-AD mice. We found that the mean vascular diameter and volume fraction in Tg-AD mice were significantly lower than those in WT mice, whereas the vessel length per volume was not significantly different (Fig. 4C). Further analysis was performed for four different sub-regions of the hippocampus excluding

those covered by the transverse hippocampal vessel and mostly by secondary vessels and capillaries, based on images of hippocampal sections similar to that shown in Fig. 4A and B (two sections per mouse for three WT mice and three Tg-AD mice). In Tg-AD mice, we found a lower mean vessel diameter and volume fraction in CA3 and DG-ml, but not in Sub and CA1–2 (Fig. 4D and E), whereas a lower mean vessel length per volume was found in CA1–2 and DG-ml, but not in Sub and CA3 (Fig. 4F). These results indicate different abnormalities of the vasculature in various sub-regions of the hippocampus in the Tg-AD mice, with DG-ml exhibiting the most severe impairment. These quantitative results are consistent with the qualitative visual impression of the vascular network in WT and Tg-AD mice (Fig. 3G and H).

### Reduced perfusion area of hippocampal vessels in Tg-AD mice

**Figure 5.**
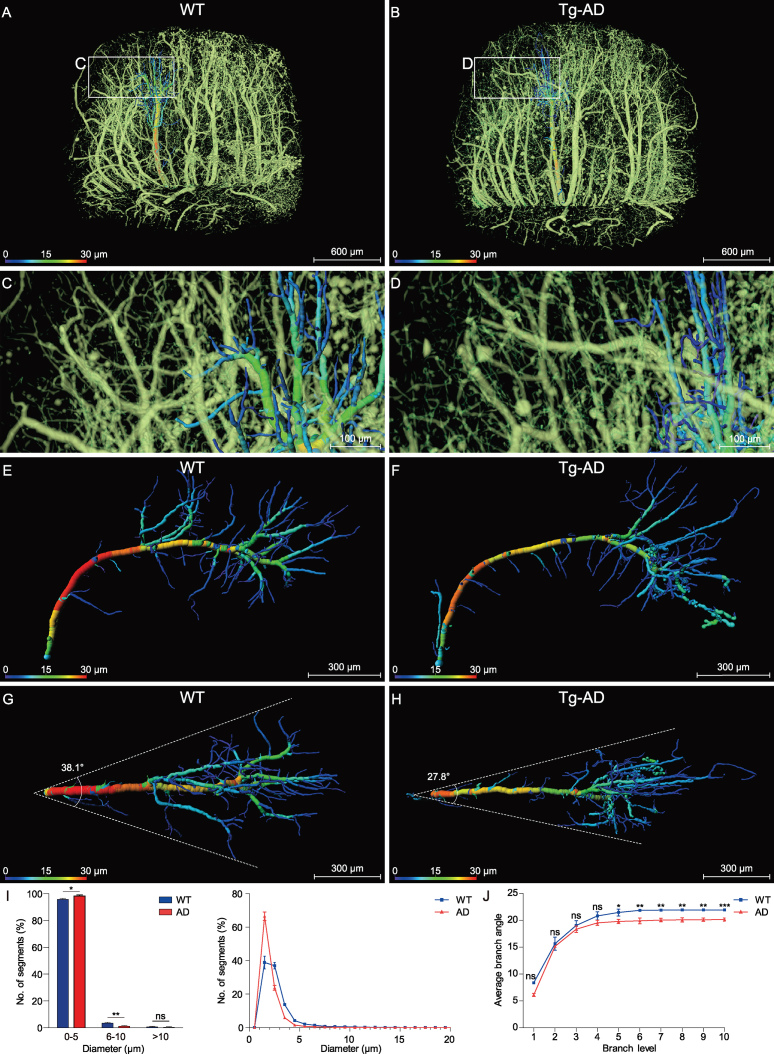
Analysis of the morphology and architecture in an individual hippocampal vessel. (A), (C), (E) and (G) WT group; (B), (D), (F) and (H) Tg-AD group. (A) and (B) The two middle hippocampal regions were extracted from the WT and Tg-AD mice in similar spatial positions. (C) and (D) Enlarged views of the boxes in (A) and (B) show the distal end of the transverse hippocampal vessels. (E) and (F) The individual hippocampal vessels were segmented manually from (A) and (B) and displayed from a lateral view, with the vessel diameters coded by colors. (G) and (H) The top view of the same vessel as shown in (E) and (F). The divergent angle of the single vascular branch is 38.1° in the WT group and 27.8° in the Tg-AD group. (I) and (J) Columns or lines in blue are the WT group; columns or lines in red are the Tg-AD group. (I) Quantitative comparison of the percentage of the number of vascular segments with different diameters (0–20 μm) between the WT and Tg-AD mice. The 98.3% number of the vascular segments was below 5 μm in the Tg-AD mice and 95.7% in the WT mice (unpaired *t*-test; *P < 0.05; **P < 0.01; ns, non-significant; *P* > 0.05; *n* = 3 mice per group). (J) Quantitative comparison of the average branching angles with different branch levels between the WT and Tg-AD mice. The average branch angles in the Tg-AD mice were significantly decreased beginning at branch level 5 (unpaired *t*-test; *P < 0.05; **P < 0.01; ***P < 0.001; ns, non-significant, *P* > 0.05; *n* = 3 mice per group). Error bars in (I) and (J) represent s.d.

To further define the vasculature impairment in Tg-AD mice, we performed further analysis on the vessel-branching pattern, by focusing on the middle hippocampus region along the longitudinal hippocampal axis (Fig. 5A and B). High-resolution images showed an apparent difference in the branching pattern, with more distal fine branches in the Tg-AD mouse (Fig. 5C and D). To further quantify the branching pattern, we traced individual vessels of the corresponding regions in the WT and Tg-AD mice. As shown by an example vessel for a WT mouse (Fig. 5E and G; at two perpendicular views) and a Tg-AD mouse (Fig. 5F and H), the individual transverse hippocampal vessel of the WT mouse exhibited regular hierarchical branching that was largely absent in the Tg-AD mouse. Vessels of small diameters (color-coded blue) were also more abundant in the Tg-AD mouse. We measured the average diameter of all vessel segments (defined as the compartment between branch nodes, three middle hippocampus regions from three mice) and found a higher percentage of segments with smaller diameters, with more than 98.3% of segments below 5 μm in the Tg-AD mice, as compared to that of the WT mice (95.7% in the WT mouse) (Fig. 5I).

As shown by the two example vessels in Fig. 5Gand H, the spatial coverage of the vascular arbors in the WT mouse was higher than that in the Tg-AD mouse. This could be attributed to the bifurcation angles of the branch node, relative to the original vessel trunk. We measured the bifurcation angles for branching at different branch levels (three middle hippocampus regions from three mice) and found a significant difference in the average angles for higher branch levels (>4) (Fig. 5J). This result is consistent with the reduced spatial coverage of individual vessels, implicating reduced blood perfusion of the hippocampal vessels associated with AD.

### Virtual endoscopic view of hippocampal vessels

**Figure 6.**
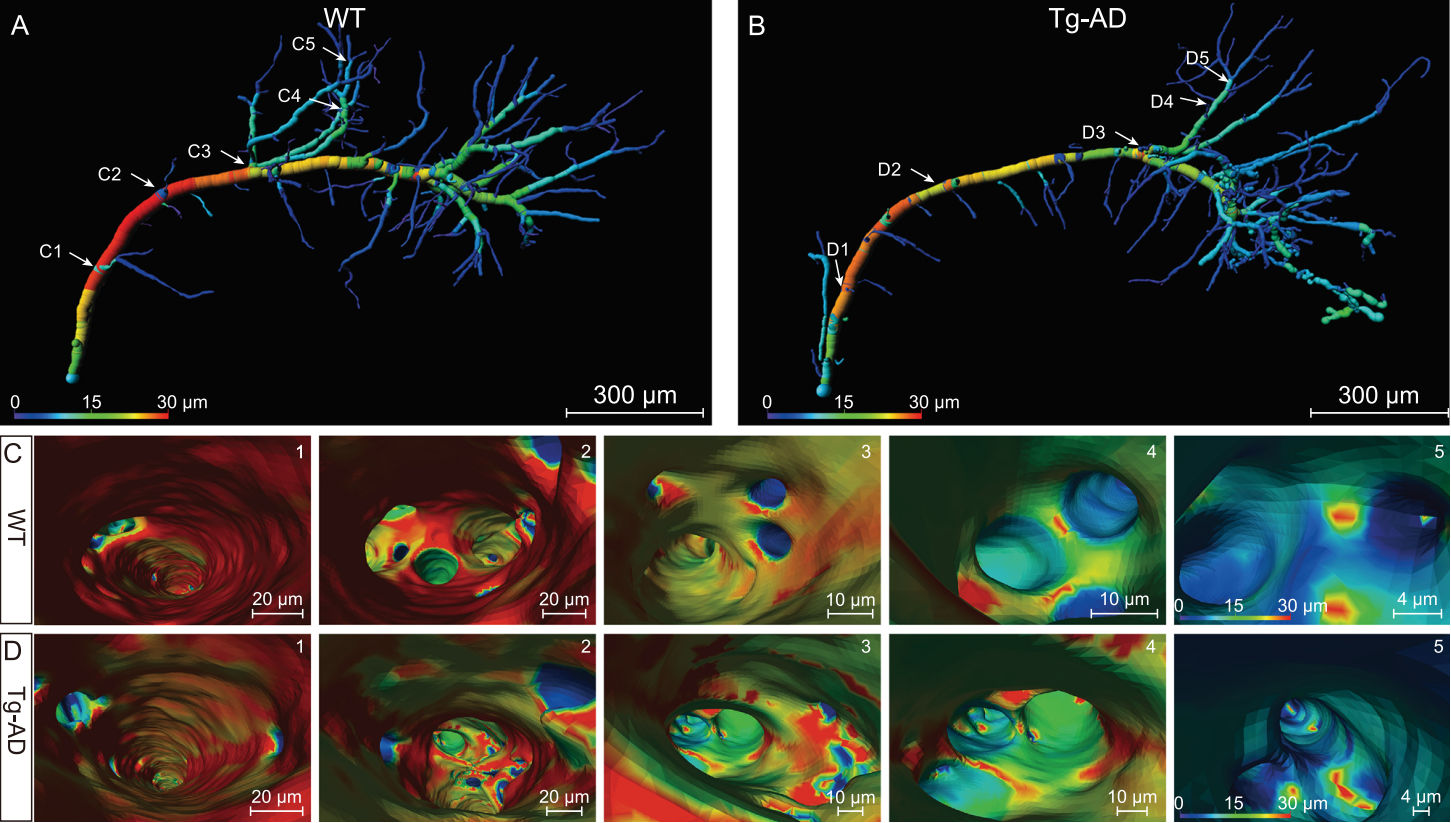
Virtual endoscopic views in the lumen of single hippocampal vessels. (A) and (B) Representative individual hippocampal vessels the same as shown in Fig. 5E and F were used to illustrate the virtual vascular endoscopy for viewing the vessel lumen, with vessel diameters coded by colors and representative viewpoints marked by the white number (C1–5 for WT mice; D1–5 for Tg-AD mice). (1–5) The virtual vascular endoscopic tracking of individual transverse hippocampal vessels in WT mice (C) and Tg-AD mice (D). (1) Initial entry into the vessels. Subsequent images along the tracking pathway are shown in (2)–(5), revealing different endovascular structures between WT and Tg-AD mice. The scale bar in each endoscopic image indicates the magnification of field of view.

An endoscopic comparison was also conducted between the WT and Tg-AD mice to investigate the inner-surface morphology and smoothness of the vascular lumen. The same two vessels as shown in Fig. 5E and F (also shown in Fig. 6A and B) were compared with virtual endoscopic views. The comparison indicates the apparent alterations of the inner-surface morphology and branch pattern in the Tg-AD mice. The color in the endoscopic images indicates the diameter of the vascular lumen. As shown in Fig. 6C and D and Supplementary Movie 3, as reflected by the color uniformity of the vessel wall, the inner surface of the vascular lumen in the Tg-AD mouse was quite rugged, while that of the WT mouse appeared much smoother. In addition, at the branch points, the lumen of the new branch opening appeared as smooth circles in the WT mouse, whereas those in the AD mouse were irregular in shape. The uneven lumen surface and irregular shape of the branch-opening sites in the Tg-AD mice are likely to affect the blood flow and microcirculation in the hippocampus.

## Discussion

In this study, we have used the advanced MOST imaging and an image-optimization method to perform visual reconstruction and quantitative analysis of the whole-mouse-brain vasculature at a high resolution, with the focus on the hippocampus. We obtained the first whole-brain vasculature atlas of both WT mice and a widely used transgenic mouse model of AD (APPswe/PSEN1dE9 mice) and provided the first quantitative characterization of vascular abnormalities in the entire hippocampus of the Tg-AD mice at micrometer resolution.

With the rapid development of imaging technology, many mesoscopic brain-wide imaging methods are beginning to facilitate research on brain structure at the cellular level. Light-sheet imaging of chemically cleared transparent whole-brain tissue without sectioning could achieve axial and lateral resolution of up to 2 μm [31] and 0.1 μm [32], respectively. Serial two-photon tomography that combines tissue-surface scanning with a two-photon microscope and serial ultra-thin sectioning allows complete whole-brain cell counts and tracing of neuronal processes, although the point-scan mode of two-photon excitation may limit the imaging speed [33]. Automatic collection of serial sections, followed by optical imaging, data registration and 3D reconstruction, were also used in methods such as Array Tomography [34] and ATUM-SEM [35]. The MOST method used here can provide tomography of a centimeter-sized whole mouse brain with axial and lateral resolution of 1.0 and 0.35 μm, respectively. This allowed us to visualize the entire brain vasculature and to identify vascular abnormalities down to capillaries. On the other hand, whole-brain imaging at micrometer resolution generates large volumes of datasets (in terabytes for each brain) and imposes the demand for effective data-processing methods, including image optimization that reduces method-dependent background noise over large dimensions, high-precision rendering of large-scale 3D images and quantitative characterization of specific structural features. In this study, we have made initial attempts in developing such data-processing methods and effective workflow for large-data mining, specifically for mesoscopic analysis of whole-brain vasculature.

Methods have been developed previously for the preprocessing of whole-brain microscopic images [26] that aimed for seamless image stitching and luminance non-uniformity correction and image-defect removal in the raw data, as well as comprehensive information extraction using the image-binarization technique [36]. In order to directly visualize the fine vasculature in the coronal images, we further developed an image-optimization method to correct the background non-uniformity and noise jamming, leading to enhanced image contrast that allowed direct visualization of somata and vessels, as well as nerve processes simultaneously at high resolution. The improved image quality and vessel continuity also allowed us to perform virtual vessel endoscopy to determine the potential lumen surface abnormity associated with AD pathology. Due to high-resolution imaging, this virtual vascular endoscopy is unique in its capability for examining the luminal morphology of blood vessels and capillaries with diameters down to 2 μm.

Vascular-distribution patterns and volumetric densities are known to be different in various brain regions [37]. The present study provides a cross-scale high-resolution 3D map of the whole-mouse-brain vasculature from large vessels to microvessels, including the smallest capillaries. Our results revealed that the hippocampus has a lower mean vascular diameter, volume fraction and length density than the cortex and thalamus, implying increased vulnerability to deficiency in blood supply. Such higher vulnerability suggested that hippocampus-dependent cognitive functions [38] are likely to be more severely affected by vasculature dysfunctions.

The 3D reconstruction of the precise hippocampal vasculature in large scale and high resolution also revealed the unique vascular network within the hippocampus, such as a rake-like distribution of many equally spaced transverse hippocampal vessels arising from longitudinal hippocampal vessels, consistently with some previous anatomical observations [39,40]. In addition, we found unique comb-like capillaries in the molecular layer of the DG, with abrupt changes in diameter and branch angle from the parent vessels that are distinct from those in other hippocampus regions. Distinct vascular distributions and originations were also found with sub-regions of the DG. Such region-specific vasculatures are consistent with the functional diversity of various regions of the hippocampus [41].

### Vasculature abnormality in Tg-AD mice

The precise alteration in the brain vasculature associated with AD remains controversial. A quantitative immunostaining and stereological analysis found no difference in the length density and capillary number between APP/PS1 mice and WT mice at 18 months [42]. However, other immunostaining studies reported a reduction in the capillary number within the corpus callosum [43] and no blood-vessel-volume change in the cortex of APP/PS1 mice [18]. In this study, we found a distinct hippocampal vascular pattern between APP/PS1 transgenic mice and WT littermates, and provided the first comprehensive description of the aberrant changes in the hippocampal vasculature, including reduced mean vessel diameter and vascular-volume fraction, particularly for the capillaries. Such a reduction in the vessel and capillary diameter and volume may directly lead to poor blood perfusion. We note that such vasculature changes were not necessarily reflected in the length density of the vessels (Fig. 4c), which was normally used in previous studies of vasculature abnormalities [42,43]. Thus, high-resolution imaging of the vasculature is necessary for revealing the precise vasculature abnormality in detail.

In addition, our study showed the reduction in the average branch angle, leading to a reduction in the blood-perfusion area in the hippocampus of Tg-AD mice. The endoscopic observation of single hippocampal vessel also revealed an uneven lumen surface in Tg-AD mice, particularly in smaller vessels and capillaries. Given the high volume fraction of the capillaries and their critical role in the normal function and survival of tissues and organs [44,45], the luminal abnormality of the capillaries and their susceptibility to damage are likely to play a major role in AD pathogenesis, causing a decreased regional CBF found in APP/PS1 mice [46]. Our results are also consistent with the previous finding that regional variation was observed in brain-capillary density [37] and metabolic alterations were affected mainly in the hippocampus and cortex [47].

Previous studies of AD pathology have revealed that the neuron loss occurs mainly in the CA1 [48–50] and adult neurogenesis in the DG is impaired [51,52]. The contribution of regional differences in vasculature dysfunction (Fig. 4D–F) to these pathologies remains to be further clarified, although the DG showed the most marked alterations in all three vasculature parameters. Our finding that the most pronounced reduction in the capillary-volume fraction occurred in the DG is consistent with the accelerated breakdown of the BBB in the DG of individuals with mild cognitive impairment [53].

In summary, our study using MOST technology [24] together with an image-optimization method led to an unprecedented understanding of the hippocampal vasculature. The capability of clear visualization and quantification of the smallest capillaries offers new avenues for research on vascular distribution and regional microcirculation over the whole brain. Our findings on the vasculature differences in normal and AD mice also offer new insights on vasculature-related AD pathogenesis and potential therapeutic approaches for AD.

## Methods

### Sample preparation and data acquisition

Three 24-month-old male APP/PS1 transgenic mice and three male WT littermates were provided and used in this work. The APP/PS1 mice carry mouse/human amyloid precursor protein with the Swedish mutation and deletion of exon 9 of human presenilin-1 [54]. The widely used mice recapitulate many AD-related phenotypes and begin to develop Aβ deposits by 6 months of age, with abundant plaques in the hippocampus and cortex by 9 months [55]. A 2-month-old male C57BL/6 mice was also involved to establish the standardized workflow in the present work. The intact brains were removed, stained and embedded using a whole-brain Nissl-staining method [26,56] that made the samples wrapped in Spurr resin (Supplementary Fig. 2A). The crucial step of the Nissl-staining method is a longer period of cardiac perfusion with phosphate-buffered saline followed by 4% paraformaldehyde (PFA) to ensure that blood vessels are absolutely empty. The hollow blood vessels could not be dyed by Nissl-staining solution, while the surrounding somata and parenchyma would be bound in different shades. Therefore, after the treatment of Nissl staining and dehydration, the hollow blood vessels were occupied by the Spurr resin (EM0300, Sigma-Aldrich). As shown in Supplementary Fig. 1D and F, the vessels were presented in higher gray values than surrounding somata and parenchyma, which rendered them easily visualized. The resin-embedded mouse-brain specimens were subsequently imaged with the MOST system (Wuhan OE-Bio Co., Ltd, Wuhan, China) to acquire raw datasets with a voxel size of 0.35 × 0.35 × 1 μm (Supplementary Fig. 2B and C). Continuous and time-consuming data acquisition lasted for more than 10 days, producing more than 12 000 coronal sections and occupying over 1.5 terabytes in the JPEG image format (Supplementary Fig. 2C). The flow chart shown in Supplementary Fig. 2 describes the overall procedure of the work.

It should be noted that the vascular diameter in our method is supposed to be smaller than that labeled by vascular endothelial specific makers. On the one hand, the vessels here are exactly the inner lumen without vascular wall. Although the innermost layer of the vascular wall is composed of elongated monolayer endothelial cells, it is necessary to take the thickness of the vascular wall into consideration when compared with the average vascular diameter in other methods. On the other hand, the samples shrank significantly after the treatment of dehydration. The estimated linear shrinkage was 25.9 ± 1.6% in the case of the isotropy of size reduction [56].

All animal procedures were performed in accordance with the National Institutes of Health Guide for the Care and Use of Laboratory Animals, under protocols approved by and strictly following the guidelines of the Institutional Animal Care and Use Committee (IACUC). The transgenic mice were obtained from The Jackson Laboratory and the corresponding WT control mice were littermates of the Tg-AD mice. The C57BL/6 mice were obtained from Shanghai SLAC Laboratory Animal Co., Ltd (Shanghai, China).

### Image-optimization-processing method

Although there is no need for data registration on these raw mass data, as the 3D global coordinate of every image tile was recorded in the file name for automatic registration [24,56], the acquired data should be preprocessed, with the aim of seamless image stitching and luminance non-uniformity correction and image-defect removal. Allowing for the redundant black-edge reserved in every image tile, the first step is to stitch the raw image tiles to achieve a coronal image sequence (Supplementary Fig. 2B and C). Moreover, the defects exist not only inside slices, but also among them, which is probably caused by potential detrimental impacts on the brain morphology and light beam, such as uneven staining in the procedure of sample preparation, incongruous illumination and altered environments of the MOST system over long periods of imaging [26,56].

A previous preprocessing program was performed for image splicing and artifacts elimination simultaneously [26]. However, it produces poor results. Taking the features of coronal images into account, an image-optimization method was designed. The specific imaging mode of MOST led to periodic strip artifacts, and the inhomogeneous staining among different brain regions resulted in uneven background and further influenced the rendering effect and morphological analysis. The optimization was implemented through steps including background correction, noise reduction and contrast enhancement. The periodic strip artifacts caused by uneven illumination were corrected by the implementation of the baseline drift-modification algorithm. The inhomogeneous staining induced non-uniform brightness was corrected according to a reference background image generated by processing the original image with an opening operator (kernel size of 75 × 75 × 75) and box filter (kernel size of 100 × 100 × 100). An edge-preserving smoothing was subsequently performed for noise reduction through a bilateral filtering algorithm with a kernel size of 3 × 3 × 3 [57]. Finally, a linear histogram transformation was applied for the contrast enhancement. The representative coronal images after the image optimization were improved markedly in background uniformity, noise reduction and image contrast (Supplementary Fig. 1B, D and F) compared to that before the image optimization (Supplementary Fig. 1A, C and E).

### Manual segmentation of the hippocampal formation

On the basis of the high-quality coronal slices, the segmentation of the hippocampal formation was carried out step by step as follows. (i) Resample the selected coronal slices including whole hippocampus to a voxel size of 3.5 × 3.5 × 10 μm using Fiji. To be convenient for reading the complete volume into the memory, it is imperative to degrade the slices of high resolution. (ii) Segment manually slice by slice with a brush to obtain contours using the segmentation editor in Amira software in reference to the Allen Reference Atlas [58] and the Franklin and Paxinos Mouse Atlas [59]. The segmentation was made in the coronal view, but the region of interest (ROI) was also visually inspected in the sagittal and horizontal sections to ensure the hippocampus was properly outlined. The delineated contours representing the borders between the interior and exterior regions of hippocampus were defined by visually comparing the specific anatomical features of every brain region to the mouse atlases mentioned above. (iii) Export the contour data to image sequence and magnify it to the original voxel size. (iv) Align the corresponding preprocessed images to labeled images to obtain segmented images using a Matlab program. (v) Surface reconstruction of the hippocampal formation in Amira software.

Manual segmentation was conducted by the same individual who was trained to use the Amira segmentation program and was proficient in brain anatomy. Only one brain sample from a 2-month-old C57BL/6 mouse was segmented bilaterally to illustrate the whole hippocampal structure and other samples were studied concentrating on the right part of the hippocampus.

### Visualization of the cerebrovascular network

Taking advantage of the volume-rendering module in Amira and the high-end graphics processing unit (GPU) card, hardware-accelerated real-time rendering was performed for the 3D visualization. The underlying model was based on the emission and absorption of light that pertains to every voxel of the viewed volume; in addition, the algorithm simulates the casting of light rays through the volume from pre-set sources. Without segmentation, the optimized data were rendered directly based on gray-value thresholding and mapped with a color-map including alpha values defining the opacity. After the optimization, the image quality had been improved apparently. The enhanced contrast made it possible to distinguish and identify the ultra-structures of vessels, neuron somata and nerve processes based on the gray difference.

The 200-μm-thick coronal slices of a 2-month-old C57BL/6 mouse containing hippocampus were selected and executed with volume rendering to visualize the complex vascular network and evaluate the performance of MOST. The other 400-μm-thick coronal slices of the same dataset were also reconstructed using maximum intensity projection (MIP) to reveal more detailed information. The colors show the relative levels of vascular densities. The blood vessels of the whole mouse hippocampus were also visualized using Amira software after getting the segmented images. We also presented a sectional view of the hippocampal vasculature, which was 200 μm in thickness and perpendicular to the long axis of the hippocampus. Then the single vascular branch was manually extracted and reconstructed from the complicated hippocampal vascular network using the Amira segmentation program. The reconstruction of the vascular network in Tg-AD and WT mice was also carried out following the technical route above. In brief, the 200-μm-thick coronal slices from the APP/PS1 and WT mice were reconstructed, respectively. The successive coronal slices were selected from the same anatomical position from the brain of these two groups. Due to the limitation of space, we merely presented the coronal view focusing on the hippocampus. And then the vasculatures of the whole hippocampus in WT and APP/PS1 mice were also visualized after obtaining the segmented images. The sectional views of the hippocampal vasculature were extracted and displayed perpendicularly to the long axis of the hippocampus. The data of WT and APP/PS1 mice were from a similar anatomical position of the hippocampus. The workflow of the visual reconstruction is displayed in Supplementary Movie 1.

### Virtual endoscopy of the single transverse hippocampal vessel

In order to explore the endoluminal views and inner surfaces of the blood vessels, the technique of virtual vascular endoscopy was introduced. As a developed post-processing technique, virtual endoscopy simulates the operation of a real fiber-optic endoscope by moving the camera along the central path in the vascular lumen. Beyond the knowledge obtained by conventional 3D reconstructions, virtual vascular endoscopy allows the viewer to explore the inner surfaces and fine internal structure from certain interesting viewpoints. To reduce the computation burden, the surface-rendering technique was equipped to calculate images resembling the views of fiber endoscopy in a real-time mode.

Based on the optimized 3D data, an individual target transverse artery has been identified and extracted manually. First, the iso-surface was generated after the segmentation was processed. Then the real-time rendering combined with camera path planning was used to simulate virtual camera recordings. Dynamically changing the position and orientation of the camera along the pathway, ‘virtual flight’ was realized for the visualization of vessels by altering the viewing angle, light source, depth encoding, shading effects and surface characteristics in the reconstructed images. For the calculation, the generated 3D-surface model was first meshed into triangular surfaces. Then, at each vertex, the module computes the distance along the vertex normal to the normal's intersection with the closest triangle of the same surface. The result is exported as a surface scalar field with a distance measure per vertex. The value generally reflects the diameter of the vessel at corresponding positions of the vertex and partially illustrates the roughness with the color changing. For example, if the color changes sharply in a small region without any branches, this indicates the rough lumen surface. The virtual vascular endoscopy enables the localization of the relevant blood vessels, visualization of the micro-structure of the vascular lumen and inner walls, and even allows measurement of the lumen diameter of selected vessels, which facilitates the assessment of the morphological and structural alternations of the inner surface and vascular-branch pattern.

### Quantification of the hippocampal vasculature

We first made a comparison of the main parameters of vascular morphology among the cortex, hippocampus and thalamus in a 2-month-old C57BL/6 mouse. Three regions of interest (similar to Fig. 1F–H) were chosen for each brain region and data are expressed as mean ± s.e.m., *n* = 1 mouse. The main morphological parameters in the vascular assessment include the mean diameter, length density, volume fraction and maximum diameter. The length density was used for measuring the total vascular length per imaging volume (meters per cubic millimeters). The volume fraction was adopted to calculate the ratio of the total vascular volume to the volume of the corresponding brain tissue.

Quantitative analyses of hippocampal vessels were conducted to determine the changes that occurred in the hippocampus of the Tg-AD mouse. We compared the mean diameter, length density and volume fraction of the whole hippocampal vessels between the WT and Tg-AD mice (*n* = 3 mice). To further quantitatively identify the vascular deformation of the brain in the Tg-AD mice, we segmented the hippocampal vasculature into several sub-regions including the subiculum (Sub), CA1–2, CA3 and molecular layer of the dentate gyrus (DG-ml) and subsequently carried out statistical analysis of these subareas. The mean diameter, length density and volume fraction of these subareas were compared between the WT and Tg-AD mice (*n* = 6 sections from three mice, two sections per mouse). Moreover, the comparison of the percentages of the number of segments with the vascular diameter changing was drawn on the middle part of the hippocampal vascular network (see Fig. 5A and B) at similar spatial positions between APP/PS1 Tg mice and WT littermates (*n* = 3 mice per group). The average branching angle was also performed to evaluate the changes accompanied by different branch levels (*n* = 3 mice per group). The ‘segment’ is defined as compartments lying between two branching nodes. The ‘branch level’ refers to a numerical structure that starts from the beginning node to the terminal node. The branch angle is the angle between the extending lines from the branch nodes. The calculation and analysis were conducted using Amira and Imaris 3D software.

### Statistical analysis

All data were analysed in GraphPad Prism 5. A two-tailed Student's *t*-test was employed to assess the statistical significance in the values between Tg-AD mice and WT littermates. Data were presented as mean ± s.e.m., or mean ± s.d., as indicated in the figure legends. A *P*-value <0.05 was considered statistically significant.

## Supplementary Material

nwz124_Supplemental_Files
