# Supplementary material for: High-resolution mapping of brain vasculature and its impairment in the hippocampus of Alzheimer's disease mice
Source: Natl Sci Rev. 2019 Aug 28;6(6):1223–38. doi: 10.1093/nsr/nwz124 (PMC8291402; doi:10.1093/nsr/nwz124)
Supplement: nwz124_Supplemental_Files [file nwz124_supplemental_files.zip › Supplementary_data.docx]

Supplementary data for

**High-resolution mapping of brain vasculature and its impairment in the hippocampus of Alzheimer’s disease mice**

Xiaochuan Zhang^1,2,†^, Xianzhen Yin^2,†^, Jingjing Zhang^2,†^, Anan Li^3,4^, Hui Gong^3,4^, Qingming Luo^3,4^, Haiyan Zhang^2,^*, Zhaobing Gao^2,^* and Hualiang Jiang^1,2,^*

Corresponding author:

Hualiang Jiang, Email: hljiang@simm.ac.cn;

Zhaobing Gao, Email: zbgao@simm.ac.cn;

Haiyan Zhang, Email: hzhang@simm.ac.cn

**This file includes:**

Supplementary Figures 1 and 2

Supplementary Movies 1, 2 and 3 (available at Link: https://pan.baidu.com/s/1nUKcHQ5VX2wGcPsixlJ0OA

Password: zzez )


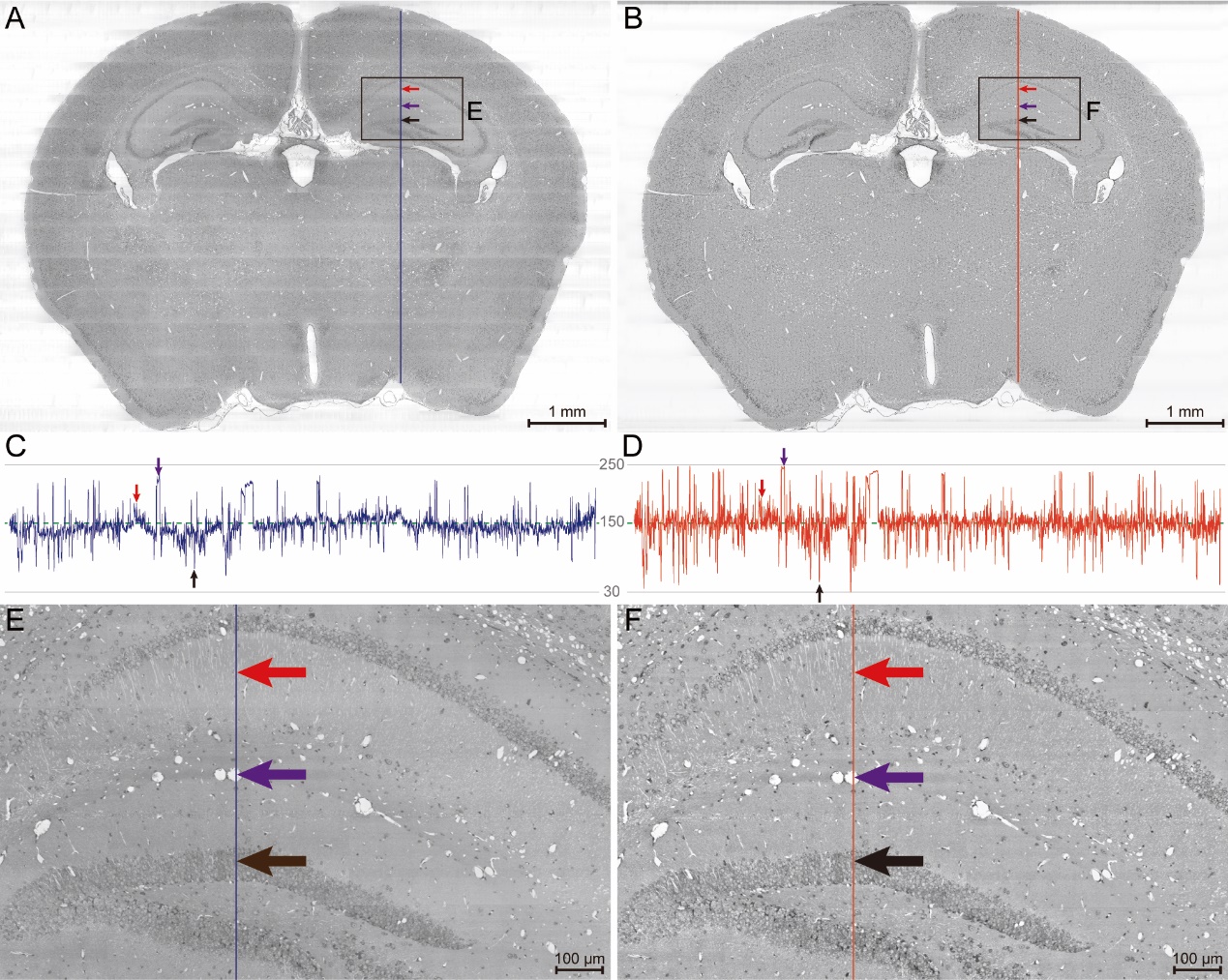


**Supplementary Figure 1. Comparison of image quality before and after** **image optimization method.**

(A-B) Representative images of coronal section show the image quality before (A) and after (B) image optimization. Red, purple and black arrows indicate the representative nerve processes, vessels and somata respectively. (A) The quality of coronal section was still influenced by stripe noise after preprocessing. (B) The image quality was significantly improved by the image optimization algorithm. (C, D) Two-dimensional graphs of the gray intensities of pixels along the drawn line within the images. The x-axis represents distance along the line and the y-axis is the gray intensity. The plot profile with blue/red color revealed the gray value along the blue/red line in A/B. The top end of lines was corresponding to the left end of the plot profile. Red, purple and black arrows denote the distinctive gray value of the representative nerve processes, vessels and somata marked in A and B. (E, F) Enlarged views of the black boxes in A and B, illustrating the nerve processes (red arrow), vessels (purple arrow) and somata (black arrow) before and after the image optimization.

**
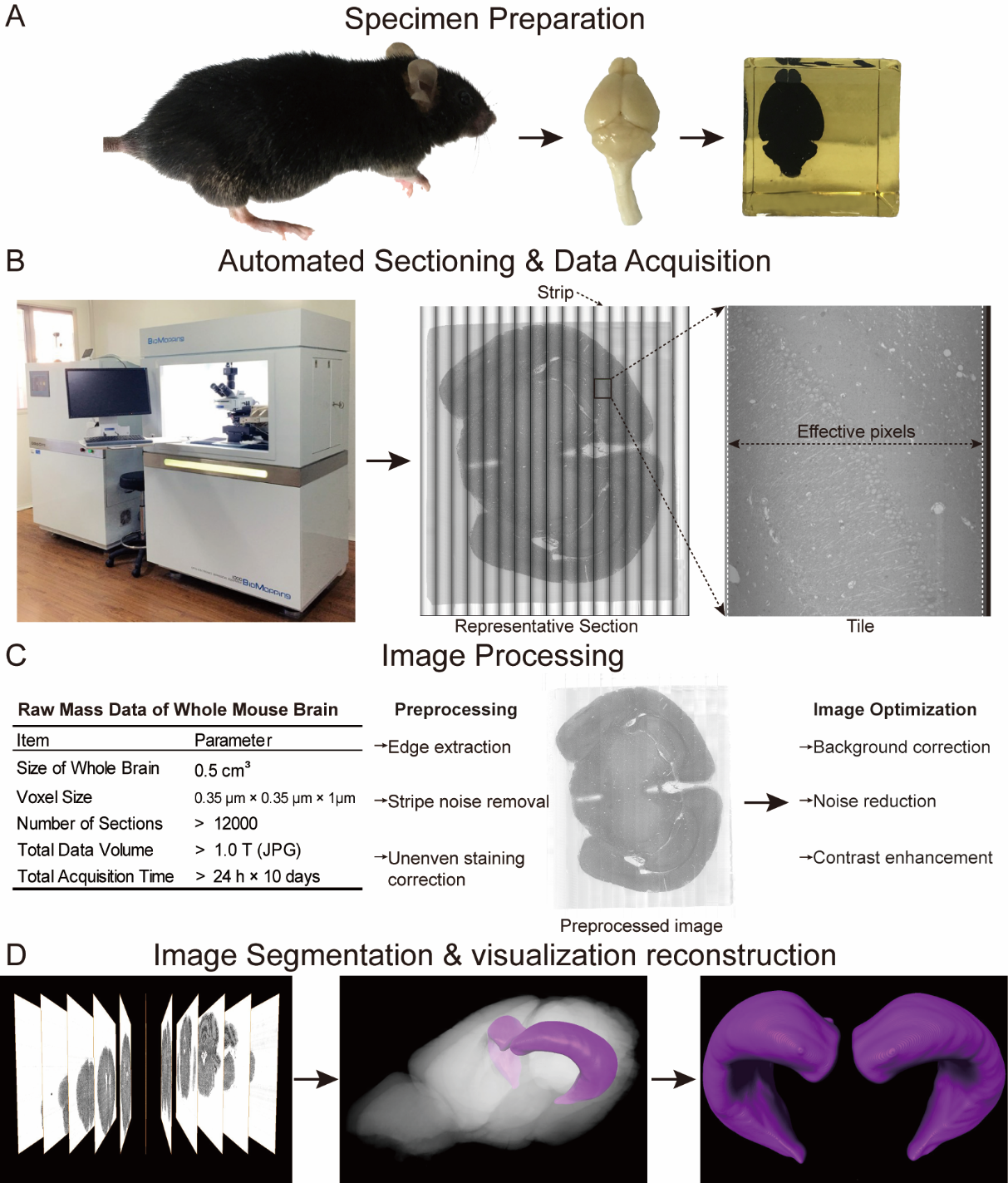
**

**Supplementary Figure 2.** **Overall workflow of the Micro-Optical Sectioning Tomography system.**

(A) Specimen preparation: The whole brains were collected after cardiac perfusion, and then the fixed brains were executed by procedures of Nissl staining and Spurr resin embedding. The embedded mouse brains look like an amber. (B) Data acquisition: The embedded brains were fastened on the base of the sectioning equipment to obtain whole brain datasets. The coronal sections were sliced into strips with regular width. Each strip was composed of many image tiles. The enlarged view of the black box shows redundant pixels reserved in each image tile and the pixels between the white dotted lines is effective. (C) Image processing: The raw mass data (the voxel size is 0.35μm × 0.35μm × 1μm) of one mouse brain contains more than 12000 coronal sections with data volume about 1.5 terabyte in JPG format. The image preprocessing is to remove redundant pixels and to solve problems with stripe noise and nonuniform brightness. After image preprocessing, the image optimization processing was conducted through steps including background correction, noise reduction and contrast enhancement. (D)Image segmentation and visualization reconstruction: Based on the high-quality image sequence, the whole mouse brain can be visualized and the hippocampus was manually segmented.

Supplementary Movies 1, 2 and 3 are available at the link below:

Link: https://pan.baidu.com/s/1nUKcHQ5VX2wGcPsixlJ0OA

Password: zzez

Supplementary Movie 1. Three-dimensional visualization and data mining workflow.

The workflow includes whole brain reconstruction, extraction of hippocampal region, visual reconstruction of vascular network, visual reconstruction of hippocampal vasculature, sectional view of hippocampal vasculature, extraction and endoscopy of single transverse hippocampal vessel.

Supplementary Movie 2. Comparison of the hippocampal vasculature between WT and Tg-AD mouse from sectional view perpendicular to the longitudinal hippocampal axis.

Supplementary Movie 3. Endoscopic comparison of single hippocampal branch between WT and Tg-AD mouse.

The inner micro-structure of vascular lumen was compared between WT and Tg-AD mice by using virtual endoscopy.
